# Fatty Acids in Habitual Diet, Plasma Phospholipids, and Tumour and Normal Colonic Biopsies in Young Colorectal Cancer Patients

**DOI:** 10.1155/2012/254801

**Published:** 2012-12-23

**Authors:** Paula Berstad, Espen Thiis-Evensen, Morten H. Vatn, Kari Almendingen

**Affiliations:** ^1^Research Centre, Akershus University Hospital, P.O. Box 95, 1478 Lørenskog, Norway; ^2^Cancer Registry of Norway, P.O. Box 5313, Majorstuen, 0304 Oslo, Norway; ^3^Section of Gastroenterology, Department of Organ Transplantation, Oslo University Hospital, Rikshospitalet, 0027 Oslo, Norway; ^4^Institute of Clinical Medicine, Faculty of Medicine, University of Oslo, Ahus Campus, 1474 Nordbyhagen, Norway; ^5^Department of Health, Nutrition, and Management, Faculty of Health Sciences, Oslo and Akershus University College of Applied Sciences, P.O. Box 4, St. Olavs Plass, 0130 Oslo, Norway; ^6^Institute of Chemistry, Biotechnology, and Food Science, Norwegian University of Life Sciences, P.O. Box 5003, 1432 Aas, Norway

## Abstract

Fatty acid metabolism is altered in colorectal cancer (CRC). We aimed to investigate incorporation of dietary *n*-6 and *n*-3 polyunsaturated fatty acids (PUFAs) into plasma phospholipids (PLs), tumour tissue, and normal mucosa in young CRC patients. We also aimed to study differences in PUFA composition between tumour and normal mucosa, and PUFA status associated with cancer stage. Sixty-five CRC patients younger than 55 years were included in a multicenter study. We assessed dietary fatty acid composition by food-frequency questionnaire. Fatty acid composition in plasma PL (*n* = 65) and tumour and normal colonic biopsies (*n* = 32) were analysed by gas chromatography. We observed a significant correlation for docosahexaenoic acid (DHA) between dietary intake and concentration in plasma PL (weight%) (*r* = 0.42; *P* = 0.001), but not for any *n*-6 PUFA. Tissue concentrations of arachidonic acid, eicosapentaenoic acid, and DHA (weight%) were 1.7–2.5 times higher in tumour than normal mucosa (*P* ≤ 0.001). Concentrations of *n*-3 and *n*-6 PUFA in plasma PL and tissues were not related to Duke's stage, although patients with more severe cancer stage reported higher intake of *n*-3 PUFA. In conclusion, we found accumulation of the long-chained *n*-3 and *n*-6 PUFA in tumour tissue in young CRC patients.

## 1. Introduction

The association between dietary fat and risk of cancer has been extensively investigated, and the composition of polyunsaturated fatty acids (PUFA) in diet seems to be of particular importance [1]. However, the effects of single dietary *n*-3 and *n*-6 PUFA, and the ratio *n*-3/*n*-6 PUFA are not completely clear. The majority of case-control studies seem to support a protective role of dietary *n*-3 PUFA and *n*-3/*n*-6 PUFA ratio [2–4], but these associations have been confirmed by only one cohort study [5], and have been contradicted or not found by several cohort studies [6–8]. Studies on serum and erythrocyte membrane fatty acid composition, regarded as biomarkers for fatty acid intake, mainly support a protective role for the very long-chained *n*-3 PUFA docosahexaenoic acid (DHA) [8–11]. Abnormalities in plasma PUFA composition may also be interpreted as metabolic changes in CRC patients [12]. Dietary and biomarker studies have not established the role of *n*-6 PUFA in CRC. Based on changes in *n*-3 and *n*-6 PUFA expression in colorectal tumours, compared to normal mucosal tissue [13, 14], also found at early stages of adenomas [13], it seems obvious that patients with CRC have an altered PUFA metabolism. Literature on CRC tumour PUFA pattern is sparse, and previous studies have not concluded on which particular PUFAs are present in diseased and normal mucosa [13, 14]. However, PUFA pattern in normal mucosal tissue seems to be similar in CRC patients and healthy subjects [13]. This indicates that the changes in mucosal fatty acid pattern are tumour and normal tissue specific in general CRC patients.

The younger CRC patients may be more genetically predisposed; their tumour might be expected to be more aggressive [15] and environmental factors relatively less pronounced in the etiology of the disease, compared to older patients. Genetic predisposition is known in patients with familial adenomatous polyposis (FAP), who may have characteristics more similar to young CRC patients. Our previous study in FAP patients showed increased concentrations of arachidonic acid (AA) and DHA, and decreased concentrations of linoleic acid (LA) and *α*-linolenic acid (ALA) in plasma phospholipids (PL) [16], opposite to the majority of findings of protective effect of DHA against development of colorectal adenomas [9–11, 17]. We therefore hypothesize that PUFA concentration in plasma phospholipid does not reflect dietary PUFA in young CRC patients. We further hypothesize that PUFA composition differs between CRC tumour tissue and normal colonic mucosal biopsies, thereby investigating if the fatty acid pattern characteristic for FAP patients could be observed in the colorectum of CRC patients. We also wanted to study whether dietary PUFA intake or concentration of PUFA in plasma PL, tumour, or normal mucosa were associated with cancer stage (Duke's stage).

## 2. Materials and Methods

### 2.1. Patients

Eighty-two patients were recruited from seven hospitals in the South-Eastern region of Norway. Inclusion criteria were adenocarcinoma of the colon or rectum and age below 55 years. Exclusion criterion was known familial syndromes affecting CRC risk including hereditary non-polyposis colorectal cancer (HNPCC) and FAP. The inclusion period was from December 2003 until April 2009.

### 2.2. Blood Sampling and Analyses

A blood sample was collected preoperatively and centrifuged within 30 min. Plasma samples were sent to Oslo University Hospital, Rikshospitalet and stored in −80°C. The frozen samples were sent on dry ice to Vitas AS, Oslo, Norway, where they were stored at −20°C until analyses. Fatty acid contribution in plasma phospholipid fraction (PL) was quantitatively determined as follows: plasma samples were thawed overnight at 4°C and vortexed for 5 sec. Dichloromethane in methanol was added to plasma and the internal standard (1,2 diheptadecaonyl-sn-glycero-3-phosphatidylcholine). After shaking and centrifugation, the supernatant was transferred into new glasses and washed in 0.9% saline solution. Lower phase was transferred to solid phase extraction columns. Polar lipids were washed out with dichloromethane/isopropanol and Methyl *tert*-butyl ether (MTBE)/formic acid. Phospholipids were eluted with methanol. After evaporation to dryness in a vacuum centrifuge, phospholipids were transmethylated by sodium methoxide, and fatty acid methyl esters (FAMEs) were extracted to hexane before gas-chromatographic (GC) analysis. The GC analysis was performed on a 7683B GC with a split/splitless injector, a 7683B automatic liquid sampler, and flame ionization detection (Agilent Technologies, Palo Alto, CA, USA). Separations were performed on a 30 m SP-2380 column (Supelco, Sigma-Aldrich, St. Louis, MO, USA).

### 2.3. Tumour and Normal Mucosa Samples and Analyses

Tumour and normal mucosa tissue samples, at least 2 × 1 × 0.5 cm in size, were collected immediately after resection of the specimen, prepared with RNA-later, transported, and stored dry in −80°C until analysis. The normal mucosa sample was taken from a normal appearing part of the resected specimen, at least 20 cm from the cancer. The frozen samples were sent on dry ice to Vitas AS, Oslo, Norway, where tissue fatty acid contribution was determined as follows: approximately 10 mg tissue was directly methylated with 3N MeOH HCl, first 30 minutes at 70°C on an ultrasonic bath, and later at 80°C on a thermoblock with 500 rpm. FAMEs were extracted with hexane, and the samples were subsequently neutralized with KOH in water. After mixing and centrifuging the hexane phase was injected into the GC-FID. The GC analysis was performed similarly as for plasma PL.

### 2.4. Dietary Intake

The patients were contacted postoperatively by telephone and invited to give dietary and lifestyle data. Dietary intake was assessed by a validated self-filled food-frequency questionnaire (FFQ) [18], designed to cover as much of the total diet as possible. Questions were related to habitual frequency of consumption and the amount of foods eaten during the one-year period prior to CRC diagnosis. Dietary supplements, such as cod liver oil, fish oil capsules, and vitamin and mineral supplements, were included. The FFQ was mailed to the participants and filled in at home. After returning of the questionnaire by mail, the participants were contacted by a dietitian by telephone, and filling of the FFQ was checked. Dietary intakes were calculated by using a database and a software system developed at the Department of Nutrition, University of Oslo (KOSTBEREGNINGSSYSTEM, version 3.2; University of Oslo, Oslo, Norway).

In the previously developed lifestyle questionnaire [19], participants were asked to describe their smoking status by detailed questions on current and lifetime smoking status. Participants were asked to assess their last weight before CRC diagnosis as well as nonvigorous and vigorous physical activity (minimum 20 min at the time). The alternative frequencies of activity were: never, less than once/week, 1-2 times/week, 3-4 times/week, 5–7 times/week, and more than 7 times/week. The participants were also asked to state their weekly hours of occupational activity prior to CRC diagnosis, and possible changes in physical activity and dietary habits after the diagnosis.

### 2.5. Ethical Approval

The study protocol was approved by the Norwegian health authorities and the regional ethics committee. The study was performed in accordance with the Helsinki Declaration. Written informed consent was obtained from all included patients.

### 2.6. Statistical Analysis

Continuous variables are expressed as median values (25th, 75th quartile). Categorical variables are presented as percentages (%). We tested differences in fatty acid concentrations between tumour and normal mucosa by paired, non-parametric Wilcoxon Signed Ranks Test, and differences in categorical variables between subgroups by chi-square statistics. We used Pearson's correlation coefficients (*r*) to test relations between variables. We also tested correlations between dietary and plasma PL fatty acids, and between PUFA in plasma PL and normal mucosa and tumour tissue in analysis stratifying by BMI (<25 and ≥25 kg/m^2^). *P*  values < 0.05 were considered as statistically significant. We conducted the statistical analysis using software SPSS 15.0 for Windows (SPSS Inc., Chicago, IL, USA). No corrections were made for multiple comparisons.

## 3. Results

### 3.1. Subject Characteristics

A total of 69 patients provided sufficient dietary data, whereas 79 patients had data on plasma PL. Totally 65 CRC patients with median age of 47 years (range 27–54 years), and median BMI of 24.8 kg/m^2^ (range 18.4–43.2 kg/m^2^), had data on fatty acid composition in diet and plasma PL. Altogether 32 patients had additional data on fatty acid composition in tumour and normal colonic mucosal tissue. Subject characteristics are given in Table 1. Forty-eight percent of the subjects were overweight or obese (≥25 kg/m^2^), whereas six percent of the patients had low BMI (<20 kg/m^2^). Thirteen percent of the subjects reported themselves as current daily smokers, whereas 37 percent reported never having smoked. First or second degree family history of CRC in 14 percent of the patients was confirmed by the Cancer Registry of Norway. The self-reported physical activity level was not associated with dietary intake of energy or fat, or BMI (not shown).

### 3.2. Dietary Fat Intake, Correlation with Plasma PL Fatty Acid Composition

Mean dietary intake of total and saturated fat was somewhat higher than in the Nordic Nutrition Recommendations [20]. Intakes of total and long-chained marine *n*-3 PUFA were in line with the recommendations (Table 2).

Dietary DHA intake and the sum of EPA and DHA intake correlated positively with the respective concentrations in plasma PL (*r* = 0.42, *P* = 0.001 and *r* = 0.36, *P* = 0.003, resp.). Other *n*-3 or *n*-6 PUFA did not show correlation between diet and plasma PL, but the ratio of *n*-3/*n*-6 PUFA showed a correlation between diet and plasma PL (*r* = 0.29, *P* = 0.02) (Table 2). The correlations did not noticeable differ between lean and overweight patients (not shown). 

### 3.3. Differences in Plasma PL Composition between Patients with High and Low Dietary EPA + DHA Intake

When separating the CRC patients into low and high dietary EPA + DHA intake at a median of 0.82 g/day, the patients with intake above the median had higher plasma PL concentration of DHA and *n*-3/*n*-6 PUFA ratio (*P* = 0.004 and *P* = 0.006, resp.), and borderline significantly lower plasma PL concentration of LA + AA and ratio of AA/EPA (*P* = 0.05 for both) (Table 3).

### 3.4. Fatty Acids in Tumour and Normal Mucosa

Median concentrations of all individual fatty acids of interest differed significantly between normal mucosa and tumour tissue (Table 4). Median concentrations of all long-chained *n*-3 and *n*-6 PUFA (C20-22) were higher in tumours than normal biopsies, whereas concentrations of the C18-chained PUFA LA and ALA were lower in tumour tissue than normal mucosa. AA and DHA were higher in tumours than normal biopsies (7.5 versus 3.5 g/100 g, *P* = 0.001, and 2.2 versus 1.3 g/100 g, *P* < 0.001, resp.). The ratio *n*-3/*n*-6 PUFA, but also the ratio AA/EPA were higher in the tumour than normal tissue (0.15 versus 0.13, *P* < 0.001 and 12.5 versus 9.7, *P* = 0.03, resp.) (Table 4).

### 3.5. Plasma PL Concentration of EPA + DHA and LA + AA Correlated to *n*-6 and *n*-3 PUFA in Tumour Tissue and Normal Mucosa

There was a significant correlation between plasma PL concentrations of EPA and DHA (weight%) and the concentrations of EPA and DHA in normal mucosa and tumour tissues. These correlations existed both for EPA and DHA separately, and for the sum of these (Table 5). Also the ratio *n*-3/*n*-6 PUFA in normal mucosa and tumour tissue significantly correlated with plasma PL concentration of EPA + DHA. The significant correlation between plasma PL concentration of EPA + DHA, and AA concentration in normal mucosa (*r* = 0.43, *P* = 0.02) remained significant only in overweight patients (*r* = 0.69, *P* = 0.005) when stratifying by BMI groups. Plasma PL concentration of LA + AA (weight%) correlated positively with LA concentration in normal mucosa tissue. There were no other correlations between plasma PL concentration of LA + AA, and tissue PUFA concentrations, but the ratio of *n*-3/*n*-6 PUFA in normal mucosa was significantly inversely correlated with plasma PL concentration of LA + AA (Table 5).

### 3.6. Duke's Stage Compared to *n*-3 and *n*-6 PUFA in Diet, Plasma PL, and Tissue

Median dietary intake of DHA was higher in CRC patients with severe Duke's stage (C + D) as compared to patients with less severe Duke's stage (A + B) (0.56 versus 0.45 g/d, *P* = 0.02) (Table 6). Further, dietary ratio of *n*-3/*n*-6 PUFA was higher in patients with Duke's stage C + D compared to A + B (0.21 versus 0.18, *P* = 0.007). There were no significant differences in dietary AA content or plasma PL concentrations of any *n*-3 or *n*-6 PUFA between the Duke's stage groups (Table 6). There was no difference between the Duke's stage groups in any of PUFA concentrations or ratios in tumour and normal mucosal tissue (not shown).

## 4. Discussion

In the present study of young CRC patients, we found that dietary intake of total PUFA and marine *n*-3 fatty acids EPA and DHA were according to national dietary recommendations. Dietary DHA intake was in this patient population related to its concentration in plasma PL, tumour tissue, and normal mucosa. Dietary intake of single *n*-6 PUFA, LA and AA did not correlate with its concentration in plasma PL, tumour tissue or normal mucosa. On the other hand, high dietary intake of EPA and DHA was reflected as high concentrations of these fatty acids and a high ratio of *n*-3/*n*-6 PUFA in plasma and tissues. Concentrations of *n*-3 and *n*-6 PUFA with chain length C20-22 (AA, EPA and DHA) and the ratio of *n*-3/*n*-6 PUFA were higher in tumour tissue than normal mucosa, whereas concentrations of PUFA with chain length C18 (LA and ALA) were higher in normal mucosa. Surprisingly, dietary DHA intake was positively related to cancer severity measured by Duke's stage.

The present results suggest that fatty acid metabolism is altered in CRC patients, as indicated by earlier studies. First, we did not find correlation between dietary intakes of single *n*-6 PUFA and plasma PL concentrations of these in CRC patients. The absence of correlation for LA between diet and plasma PL agrees with an earlier similar finding in FAP patients [16], but disagrees with large studies in average, diverse adult population, which have demonstrated a significant positive correlation between LA concentration in plasma PL (weight%) and habitual diet assessed by FFQ [21, 22]. Another sign of abnormal LA metabolism in CRC in the present study was that concentration of LA in plasma PL significantly correlated with LA concentration in normal mucosal tissue, but not in tumour tissue. This indicates an altered incorporation of LA from plasma PL into tumour tissue, or increased elongation of LA into AA in tumours. As a contrast, there were high correlations between intake levels, plasma PL and tumour and normal mucosal tissue concentrations for EPA and DHA in the present CRC patient population. Such high correlations between intake and biomarkers of these external PUFA are found in average population [21, 22] and were expected also for LA. *n*-3 PUFA concentrations in plasma PL were reflected in their concentrations in both normal mucosa and tumour tissue in the present study. An unexplainable finding was that *n*-3 PUFA concentration in plasma PL correlated also with AA concentration in normal mucosa, and only in overweight and not in lean patients.

The second major finding on altered fat metabolism in CRC patients was that PUFA concentrations of CRC tumour tissue differed from non-diseased mucosal tissue in the present patient group; AA, EPA, and DHA were more abundant in tumour tissue than in normal colonic mucosa. This reflects the PUFA pattern in plasma PL found earlier in FAP patients as compared to control subjects [16]. Similar results on increased AA and DHA concentrations in human CRC tumours [14] and a higher concentration of AA in rat tumour tissue [23] have been found. In contrast, the opposite has also been found; lower AA concentration in phospholipids extracted from human colon cancer cells compared to normal mucosa cells [24], non-different concentrations of AA and DHA, and lower concentration of EPA in human diseased versus normal mucosa [13]. The ratio *n*-3/*n*-6 PUFA in plasma PL and tumour tissue was in the present study correlated to dietary intake of EPA and DHA and was higher in tumour tissue than in normal mucosa. This suggests that EPA and DHA are incorporated from diet into plasma and tissues in CRC patients, but that these are particularly accumulated in tumour tissue. The lower concentrations of ALA in tumour than normal mucosal tissue may also suggest an increased elongation of ALA into EPA and DHA in tumours. The markedly increased AA concentration and lower LA concentration may further indicate an increased elongation of LA into AA in tumour tissue. Concentrations of all other measured fatty acids were also significantly different between tumour tissue and normal mucosa. Interpretation of differences in SFA and MUFA concentrations is difficult and may reflect the differences in PUFA concentrations.

The third and most unexplainable finding of altered PUFA metabolism in the present study was that patients with the most advanced cancer stage reported highest dietary intake of DHA, and ratio of dietary *n*-3/*n*-6 PUFA. However, this difference in intake between Duke's stage groups A + B and C + D was not reflected in plasma PL. One reasonable explanation might be overreporting of dietary marine *n*-3 PUFA intake among those with the most severe diagnosis. The FFQ was filled by the patients up to four weeks after the CRC diagnosis. Dietary habits may have changed after the diagnosis, biasing reporting of average diet before the diagnosis. A possible true relationship between higher dietary intake of DHA and CRC severity would be unexpected in the light of the major evidence for a protecting effect of *n*-3 PUFA in colorectal carcinogenesis [25, 26]. Animal and cell line experiments suggest that *n*-3 PUFA on the contrary may reduce colorectal tumour formation [27] and suppress AA-induced proliferation in colon carcinoma cells [28]. There also is a plausible pathway for the anticarcinogenic effect of *n*-3 PUFA through inhibiting PGE_2_ synthesis and consequently reducing COX-2 expression [29, 30], an important step in colorectal carcinogenesis. Further, there is evidence that dietary *n*-3 PUFA intake protects against inflammation in cancer patients by an effect on COX-2 levels [31, 32]. Only very few reports of procarcinogenic effects of *n*-3 PUFA can be found [33]. A possible carcinogenic effect of dietary EPA and DHA might relate to proinflammatory activity of these highly oxidizable fatty acids. High-dose *n*-3 PUFA intervention (2.5–4.8 g EPA + DHA per day) in other patient groups has resulted in increased concentrations of soluble inflammatory markers of endothelial function [34, 35]. These, as well as other inflammatory markers have been related to CRC development and increasing Duke's stage [36–38]. The median dietary intake of EPA and DHA in the present patient group was, however, according to national recommendations, and only two patients reported intake levels above 2.0 g per day. In spite of an average intake of fatty acids as recorded by the questionnaires, overweight was prevalent (48% of the patients with BMI ≥25 kg/m^2^). Malignancy at younger age may indicate genetic involvement, but also a direct relationship between overweight and colorectal neoplasia could be possible [39]. Nevertheless, we did not observe higher prevalence of overweight or obesity in the group with higher Duke's stage. Future long term studies are needed to confirm a possible association between intake of high-dose *n*-3 PUFA intake and Duke's stage.

Lack of a control group is a weakness of the present study. The study would also have been strengthened by additionally analyzing fatty acid patterns in adenomas, since colorectal neoplasia develops over many years and the metabolic situation in cancerous tissue is different from that in adenomas. Further, it is unclear to what extent fatty acid intake right before the diagnosis, as assessed in the present study, is the critical period of intake with regard to tumorigenesis. The most optimal study design might therefore include dietary intake, plasma and tissue samples from patients with colorectal adenomas, and CRC patients and healthy controls. 

## 5. Conclusions

The present study showed that metabolism of *n*-6 and *n*-3 PUFA was altered in young CRC patients. Dietary LA did not correlate with its concentration in plasma PL and normal tissue, indicating an increased elongation of LA into AA in tumour tissue. The higher concentrations of AA, EPA and DHA, and ratio of *n*-3/*n*-6 PUFA in colorectal tumours than in normal mucosal tissue warrants further investigations. We suggest that future prospective studies address the possible relation between dietary intake of *n*-3 PUFA and Duke's stage in CRC in comparison to normal mucosa and adenomas, both among young and old patients. 

## Figures and Tables

**Table 1:** Characteristics of the colorectal cancer patients (*n*=65)^a^.

	*n* (%)
Male	35 (54)
Age categories (years)	
<40	12 (19)
40–49	30 (46)
50–54	23 (35)
BMI categories (kg/m^2^)^b^	
<20.0	4 (6)
20.0–24.9	30 (46)
25.0–29.9	24 (37)
30.0+	7 (11)
Smoking status^c^	
Never	23 (37)
Ex and occasional	31 (50)
Current	8 (13)
Physical activity^c^	
Low	18 (29)
Middle	28 (45)
High	16 (26)
1st or 2nd degree family history of CRC	9 (14)
Duke's stage	
A	5 (8)
B	22 (34)
C	19 (29)
D	19 (29)

^a^ Abbreviationsare as follows: BMI: body mass index (kg/m^2^); CRC: colorectal cancer.

^b^Three subjects had self-reported height and weight.

^c^Missing value in three subjects (*n* = 62).

**Table 2:** Median (25th, 75th quartile) dietary intake of fatty acids (g/day and *E*%) and fatty acid concentrations of plasma phospholipids (PL) (weight%), Nordic Nutrition Recommendations (NRR) and correlation between diet (g/day) and plasma PL in colorectal cancer patients (*n* = 65)^a^.

	Dietary fat intake		NRR	Plasma PL (weight %)	*r*
	g/day	*E*%			
Total fat	90.3 (75.7, 107.5)	33.8 (30.4, 36.9)	<30 *E*%		
Total saturated fatty acids	33.7 (28.3, 41.1)	12.9 (11.0, 14.0)	<10 *E*%	40.2 (39.5, 40.9)	0.23
16:0 (palmitic acid)	16.9 (14.0, 19.4)	6.3 (5.4, 6.9)		27.5 (26.4, 28.9)	0.20
18:0 (stearic acid)	7.9 (6.7, 10.1)	3.1 (2.7, 3.5)		12.5 (11.9, 13.5)	−0.05
16:1*n*-7 (palmitoleic acid)	1.6 (1.4, 1.8)	0.6 (0.5, 0.7)		0.52 (0.46, 0.69)	0.31^c^
18:1*n*-9 (oleic acid)	27.3 (22.0, 32.6)	10.1 (8.5, 11.4)		8.5 (7.8, 9.4)	0.20^b^
20:1*n*-9 (eicosenoic acid)	0.7 (0.6, 0.9)	0.3 (0.2, 0.3)		0.14 (0.12, 0.16)	0.12
18:2*n*-6 (linoleic acid) (LA)	15.2 (12.1, 19.5)	5.7 (4.7, 6.7)		20.3 (17.4, 22.0)	0.10
18:3*n*-3 (α-linolenic acid (ALA)	2.1 (1.8, 2.8)	0.8 (0.7, 0.9)		0.25 (0.21, 0.30)	0.12
20:4*n*-6 (arachidonic acid) (AA)	0.14 (0.11, 0.18)	0.05 (0.04, 0.06)		9.2 (7.9, 10.7)	0.03
20:5*n*-3 (eicosapentaenoic acid) (EPA)	0.30 (0.21, 0.43)	0.1 (0.07, 0.18)		1.3 (1.0, 2.2)	0.21
22:6*n*-3 (docosahexaenoic acid) (DHA)	0.50 (0.38, 0.64)	0.2 (0.13, 0.27)		5.6 (4.7, 6.9)	0.42^d^
Sum EPA + DHA	0.82 (0.60, 1.04)	0.3 (0.2, 0.4)	≥0.45 g/day	7.2 (5.8, 9.2)	0.36^c^
Ratio AA/EPA	0.42 (0.35, 0.67)			6.9 (4.8, 9.1)	0.14
Sum *n*-3 fatty acids (ALA + EPA + DHA)	3.08 (2.55, 3.77)	1.1 (1.0, 1.3)	>1 *E*%	7.35 (6.11, 9.38)	0.16
Sum *n*-6 fatty acids (LA + AA)	15.37 (12.30, 19.80)	5.7 (4.8, 6.8)		30.10 (27.55, 31.65)	−0.08
Ratio *n*-3/*n*-6 fatty acids	0.19 (0.17, 0.23)			0.25 (0.20, 0.33)	0.29^b^
Total polyunsaturated fatty acids	18.4 (14.9, 23.4)	7.0 (6.0, 8.0)	5–10 *E*%	37.7 (36.5, 39.2)	−0.16

^a^Abbreviations are as follows: *E*%: percent of energy intake; NRR: Nordic Nutrition Recommendations; PL: phospholipids; *r*: Pearson correlation coefficient.

^b^Significant correlation (*P* < 0.05).

^c^Significant correlation (*P* ≤ 0.01).

^d^Significant correlation (*P* = 0.001).

**Table 3:** Median (25th, 75th quartile) fatty acid concentrations of plasma phospholipids (PL) (weight%) in colorectal cancer patients separated by low and high dietary daily EPA + DHA intake (<0.82 and 0.82+ g/day) (*n* = 65)^a^.

Fatty acids in plasma PL (weight%)	Subjects with dietary EPA + DHA intake <0.82 g/day (*n* = 32)	Subjects with dietary EPA + DHA intake 0.82+ g/day (*n* = 33)	*P* value
18:2*n*-6 (LA)	21.1 (18.4, 23.6)	19.5 (17.3, 21.4)	0.12
20:4*n*-6 (AA)	9.4 (7.8, 10.4)	8.9 (7.9, 11.3)	0.96
Sum LA + AA	31.3 (28.7, 32.5)	29.1 (27.5, 31.1)	0.05
20:5*n*-3 (EPA)	1.2 (1.0, 1.6)	1.6 (1.1, 2.3)	0.06
22:6*n*-3 (DHA)	5.1 (4.4, 6.3)	6.3 (5.5, 7.6)	0.004
Sum EPA + DHA	6.4 (5.6, 7.7)	7.8 (6.8, 9.6)	0.006
Ratio AA/EPA	7.7 (5.7, 9.6)	5.5 (4.1, 8.0)	0.05
Ratio *n*-3/*n*-6 fatty acids	0.21 (0.18, 0.27)	0.27 (0.23, 0.35)	0.006

^a^Abbreviations are as follows: AA: arachidonic acid; DHA: docosahexaenoic acid; EPA: eicosapentaenoic acid; LA: linoleic acid; PL: phospholipids.

**Table 4:** Median (25th, 75th quartile) fatty acid concentrations (weight%) in normal duodenal mucosa and tumour biopsies in colorectal cancer patients (*n* = 32).

	Normal mucosa (weight%)	Tumour tissue (weight%)	*P* value
16:0 (palmitic acid)	20.0 (18.6, 21.6)	18.3 (17.9, 19.6)	0.001
18:0 (stearic acid)	8.1 (5.9, 10.3)	12.5 (11.0, 13.4)	<0.001
16:1*n*-7 (palmitoleic acid)	3.2 (1.9, 4.6)	1.8 (1.4, 2.5)	0.007
18:1*n*-9 (oleic acid)	33.6 (29.2, 39.6)	23.1 (21.2, 24.8)	<0.001
18:2*n*-6 (linoleic acid) (LA)	13.5 (12.1, 15.5)	12.2 (10.8, 13.4)	0.001
18:3*n*-3 (*α*-linolenic acid (ALA)	0.59 (0.43, 0.76)	0.25 (0.15, 0.29)	<0.001
20:4*n*-6 (arachidonic acid) (AA)	3.5 (1.8, 6.4)	7.5 (6.1, 8.2)	0.001
20:5*n*-3 (eicosapentaenoic acid) (EPA)	0.23 (0.17, 0.52)	0.57 (0.32, 0.85)	0.001
22:6*n*-3 (docosahexaenoic acid) (DHA)	1.30 (0.93, 1.59)	2.15 (1.67, 2.64)	<0.001
Sum EPA + DHA	1.5 (1.1, 2.1)	2.7 (2.1, 3.4)	<0.001
Ratio AA/EPA	9.7 (7.9, 17.3)	12.5 (8.5, 20.1)	0.03
Sum *n*-3 fatty acids (ALA + EPA + DHA)	2.2 (1.8, 2.7)	2.9 (2.3, 3.6)	<0.001
Sum *n*-6 fatty acids (LA + AA)	16.7 (15.2, 21.1)	19.8 (17.2, 20.5)	0.11
Ratio *n*-3/*n*-6 fatty acids	0.13 (0.11, 0.15)	0.15 (0.12, 0.18)	<0.001
Sum polyunsaturated fatty acids	18.5 (17.2, 23.6)	23.0 (19.3, 24.4)	0.05

**Table 5:** Correlation between selected polyunsaturated fatty acids and fatty acid ratios in tumour and normal mucosal biopsies and plasma phospholipids (PL) EPA + DHA and LA + AA concentrations (weight%) in colorectal cancer patients (*n* = 32)^a^.

	*r*	
	normal mucosa (*n* = 32)	tumour tissue (*n* = 32)
EPA + DHA concentration in plasma PL correlated with tissue concentration of		
18:2*n*-6 (LA)	−0.30	0.05
20:4*n*-6 (AA)	0.43^b^	−0.05
Sum LA + AA	0.15	0.001
20:5*n*-3 (EPA)	0.44^c^	0.51^c^
22:6*n*-3 (DHA)	0.71^d^	0.44^c^
Sum EPA + DHA	0.66^d^	0.50^c^
Ratio AA/EPA	−0.07	−0.55^d^
Ratio *n*-3/*n*-6 fatty acids	0.77^d^	0.70^d^
LA + AA concentration in plasma PL correlated with tissue concentration of		
18:2*n*-6 (LA)	0.50^c^	0.12
20:4*n*-6 (AA)	−0.22	0.24
Sum LA + AA	0.14	0.25
20:5*n*-3 (EPA)	−0.17	−0.20
22:6*n*-3 (DHA)	−0.33	−0.18
Sum EPA + DHA	−0.29	−0.20
Ratio AA/EPA	−0.07	0.36^b^
Ratio *n*-3/*n*-6 fatty acids	−0.47^c^	−0.42^b^

^a^Abbreviations are as follows: AA: arachidonic acid; DHA: docosahexaenoic acid; EPA: eicosapentaenoic acid; LA: linoleic acid; PL: phospholipids; *r*: Pearson correlation coefficient.

^b^Significant correlation (*P* < 0.05).

^c^Significant correlation (*P* ≤ 0.01).

^d^Significant correlation (*P* = 0.001).

**Table 6:** Median (25th, 75th quartile) content of selected polyunsaturated fatty acids and ratio *n*-3/*n*-6 fatty acids in diet and plasma phospholipids (PL) in colorectal cancer patients, separated by Dukes stage A + B and C + D (*n* = 65)^a^.

	Dukes stage		
	A + B (*n* = 27)	C + D (*n* = 38)	*P* value
Diet (g/day)			
Dietary 20:4*n*-6 (AA)	0.13 (0.11, 0.15)	0.15 (0.11, 0.20)	0.13
Dietary 20:5*n*-3 (EPA)	0.26 (0.18, 0.37)	0.34 (0.24, 0.53)	0.10
Dietary 22:6*n*-3 (DHA)	0.45 (0.33, 0.58)	0.56 (0.44, 0.94)	0.02
Sum dietary EPA + DHA	0.72 (0.50, 0.94)	0.88 (0.68, 1.49)	0.04
Ratio dietary *n*-3/*n*-6 fatty acids	0.18 (0.16, 0.21)	0.21 (0.18, 0.27)	0.007
Plasma PL (g/100 g)			
Plasma PL 20:4*n*-6 (AA)	9.2 (7.7, 10.8)	9.3 (8.0, 10.5)	0.85
Plasma PL 20:5*n*-3 (EPA)	1.2 (1.0, 1.7)	1.5 (1.1, 2.2)	0.30
Plasma PL 22:6*n*-3 (DHA)	5.4 (4.4, 6.7)	6.0 (5.0, 7.2)	0.20
Sum plasma PL EPA + DHA	6.7 (5.8, 7.7)	7.6 (6.0, 9.4)	0.17
Ratio plasma PL *n*-3/*n*-6 fatty acids	0.25 (0.18, 0.28)	0.26 (0.20, 0.35)	0.32

^a^Abbreviation is as follows: PL: phospholipids.

## References

[1] Nkondjock A, Shatenstein B, Maisonneuve P, Ghadirian P (2003). Specific fatty acids and human colorectal cancer: an overview. *Cancer Detection and Prevention*.

[2] Kato I, Majumdar AP, Land SJ, Barnholtz-Sloan JS, Severson RK (2010). Dietary fatty acids, luminal modifiers, and risk of colorectal cancer. *International Journal of Cancer*.

[3] Kim S, Sandler DP, Galanko J, Martin C, Sandler RS (2010). Intake of polyunsaturated fatty acids and distal large bowel cancer risk in whites and African Americans. *American Journal of Epidemiology*.

[4] Theodoratou E, McNeill G, Cetnarskyj R (2007). Dietary fatty acids and colorectal cancer: a case-control study. *American Journal of Epidemiology*.

[5] Murff HJ, Shu XO, Li H (2009). A prospective study of dietary polyunsaturated fatty acids and colorectal cancer risk in Chinese women. *Cancer Epidemiology, Biomarkers & Prevention*.

[6] Butler LM, Wang R, Koh WP, Stern MC, Yuan JM, Yu MC (2009). Marine n-3 and saturated fatty acids in relation to risk of colorectal cancer in Singapore Chinese: a prospective study. *International Journal of Cancer*.

[7] Daniel CR, McCullough ML, Patel RC (2009). Dietary intake of *ω*-6 and *ω*-3 fatty acids and risk of colorectal cancer in a prospective cohort of U.S. men and women. *Cancer Epidemiology, Biomarkers & Prevention*.

[8] Geelen A, Schouten JM, Kamphuis C (2007). Fish consumption, n-3 fatty acids, and colorectal cancer: a meta-analysis of prospective cohort studies. *American Journal of Epidemiology*.

[9] Kojima M, Wakai K, Tokudome S (2005). Serum levels of polyunsaturated fatty acids and risk of colorectal cancer: a prospective study. *American Journal of Epidemiology*.

[10] Kuriki K, Wakai K, Hirose K (2006). Risk of colorectal cancer is linked to erythrocyte compositions of fatty acids as biomarkers for dietary intakes of fish, fat, and fatty acids. *Cancer Epidemiology, Biomarkers & Prevention*.

[11] Pot GK, Geelen A, van Heijningen EM, Siezen CLE, van Kranen HJ, Kampman E (2008). Opposing associations of serum n-3 and n-6 polyunsaturated fatty acids with colorectal adenoma risk: an endoscopy-based case-control study. *International Journal of Cancer*.

[12] Baro L, Hermoso JC, Nunez MC, Jimenez-Rios JA, Gil A (1998). Abnormalities in plasma and red blood cell fatty acid profiles of patients with colorectal cancer. *British Journal of Cancer*.

[13] Fernández-Bañares F, Esteve M, Navarro E (1996). Changes of the mucosal n3 and n6 fatty acid status occur early in the colorectal adenoma-carcinoma sequence. *Gut*.

[14] Neoptolemos JP, Husband D, Imray C, Rowley S, Lawson N (1991). Arachidonic acid and docosahexaenoic acid are increased in human colorectal cancer. *Gut*.

[15] Hemminki K, Santi I, Weires M, Thomsen H, Sundquist J, Bermejo JL (2010). Tumor location and patient characteristics of colon and rectal adenocarcinomas in relation to survival and TNM classes. *BMC Cancer*.

[16] Almendingen K, Høstmark AT, Fausa O, Mosdøl A, Aabakken L, Vatn MH (2007). Familial adenomatous polyposis patients have high levels of arachidonic acid and docosahexaenoic acid and low levels of linoleic acid and *α*-linolenic acid in serum phospholipids. *International Journal of Cancer*.

[17] Ghadimi R, Kuriki K, Tsuge S (2008). Serum concentrations of fatty acids and colorectal adenoma risk: a case-control study in Japan. *Asian Pacific Journal of Cancer Prevention*.

[18] Andersen LF, Veierød MB, Johansson L, Sakhi A, Solvoll K, Drevon CA (2005). Evaluation of three dietary assessment methods and serum biomarkers as measures of fruit and vegetable intake, using the method of triads. *British Journal of Nutrition*.

[19] Almendingen K, Fausa O, Høstmark AT (2009). Serum nutrients and habitual dietary intake in colectomized FAP patients in Norway. *European Journal of Nutrition*.

[20] Becker W, Alexander J, Andersen S (2006). Nordic nutrition recommendations. *Ugeskrift for Læger*.

[21] Hodge AM, Simpson JA, Gibson RA (2007). Plasma phospholipid fatty acid composition as a biomarker of habitual dietary fat intake in an ethnically diverse cohort. *Nutrition, Metabolism and Cardiovascular Diseases*.

[22] Thiebaut ACM, Rotival M, Gauthier E (2009). Correlation between serum phospholipid fatty acids and dietary intakes assessed a few years earlier. *Nutrition and Cancer*.

[23] Nicholson ML, Neoptolemos JP, Clayton HA, Talbot IC, Bell PRF (1991). Increased cell membrane arachidonic acid in experimental colorectal tumours. *Gut*.

[24] Oraldi M, Trombetta A, Biasi F, Canuto RA, Maggiora M, Muzio G (2009). Decreased polyunsaturated fatty acid content contributes to increased survival in human colon cancer. *Journal of Oncology*.

[25] Cockbain AJ, Toogood GJ, Hull MA (2011). Omega-3 polyunsaturated fatty acids for the treatment and prevention of colorectal cancer. *Gut*.

[26] Murff HJ, Shrubsole MJ, Cai QY (2012). Dietary intake of PUFAs and colorectal polyp risk. *The American Journal of Clinical Nutrition*.

[27] Kenar L, Karayilanoglu T, Aydin A, Serdar M, Kose S, Erbil MK (2008). Protective effects of diets supplemented with omega-3 polyunsaturated fatty acids and calcium against colorectal tumor formation. *Digestive Diseases and Sciences*.

[28] Habbel P, Weylandt KH, Lichopoj K (2009). Docosahexaenoic acid suppresses arachidonic acid-induced proliferation of LS-174T human colon carcinoma cells. *World Journal of Gastroenterology*.

[29] Bagga D, Wang L, Farias-Eisner R, Glaspy JA, Reddy ST (2003). Differential effects of prostaglandin derived from *ω*-6 and *ω*-3 polyunsaturated fatty acids on COX-2 expression and IL-6 secretion. *Proceedings of the National Academy of Sciences of the United States of America*.

[30] Calviello G, Di Nicuolo F, Gragnoli S (2004). n-3 PUFAs reduce VEGF expression in human colon cancer cells modulating the COX-2/PGE2 induced ERK-1 and -2 and HIF-1*α* induction pathway. *Carcinogenesis*.

[31] Fradet V, Cheng L, Casey G, Witte JS (2009). Dietary omega-3 fatty acids, cyclooxygenase-2 genetic variation, and aggressive prostate cancer risk. *Clinical Cancer Research*.

[32] van der Meij BS, Langius JAE, Smit EF (2010). Oral nutritional supplements containing (n-3) polyunsaturated fatty acids affect the nutritional status of patients with stage III non-small cell lung cancer during multimodality treatment. *The Journal of Nutrition*.

[33] Akedo I, Ishikawa H, Nakamura T (1998). Three cases with familial adenomatous polyposis diagnosed as having malignant lesions in the course of a long-term trial using
docosahexanoic acid (DHA)-concentrated fish oil capsules. *Japanese Journal of Clinical Oncology*.

[34] Johansen O, Seljeflot I, Høstmark AT, Arnesen H (1999). The effect of supplementation with omega-3 fatty acids on soluble markers of endothelial function in patients with coronary heart disease. *Arteriosclerosis, Thrombosis, and Vascular Biology*.

[35] Seljeflot I, Arnesen H, Brude IR, Nenseter MS, Drevon CA, Hjermann I (1998). Effects of omega-3 fatty acids and/or antioxidants on endothelial cell markers. *European Journal of Clinical Investigation*.

[36] Ferroni P, Roselli M, Martini F (2004). Prognostic value of soluble P-selectin levels in colorectal cancer. *International Journal of Cancer*.

[37] Holubec L, Topolcan O, Finek J (2005). Markers of cellular adhesion in diagnosis and therapy control of colorectal carcinoma. *Anticancer Research*.

[38] Roselli M, Guadagni F, Martini F (2003). Association between serum carcinoembryonic antigen and endothelial cell adhesion molecules in colorectal cancer. *Oncology*.

[39] Almendingen K, Hofstad B, Vatn MH (2001). Does high body fatness increase the risk of presence and growth of colorectal adenomas followed up in situ for 3 years?. *American Journal of Gastroenterology*.

